# Saddle pulmonary embolism in the setting of COVID-19 infection: A systematic review of case reports and case series

**DOI:** 10.1515/med-2023-0724

**Published:** 2023-06-01

**Authors:** Hassan Choudry, Fateen Ata, Wanis Ibrahim, Mohammad Omer Rehman Rana, Shoaib Ahmad, Asim Mehmood, Basir Afzaal Gill, Mahammed Khan Suheb

**Affiliations:** Department of Respiratory Medicine, University Hospital of Leicester, Leicester LE1, UK; Department of Endocrinology, Hamad General Hospital, Hammad Medical Corporation, PO BOX 3050, Doha, Qatar; Department of Internal Medicine and Pulmonology, Hammad Medical Corporation, Doha, Qatar; Department of Adult Cardiology, Chaudhary Pervaiz Ilahi Institute of Cardiology, Wazirabad, Pakistan; Department of Medicine, Punjab Medical College, Faisalabad, Pakistan; Respiratory Department, Derriford Hospital, University Hospitals Plymouth, Plymouth, UK; Intensive Care Unit, Department of Anaesthesia, Jinnah Hospital, Lahore, Pakistan; Critical Care Department, St. Luke’s Aurora Hospital, Milwaukee, Wisconsin, USA

**Keywords:** saddle pulmonary embolism, SPE, COVID-19, coronavirus disease 2019, SARS-CoV-2, severe acute respiratory syndrome-related coronavirus 2

## Abstract

Saddle pulmonary embolism (SPE) is a rare type of pulmonary embolism that can lead to hemodynamic compromise causing sudden deaths. Due to a dearth of large prospective studies in this area, little is known regarding the epidemiology, and prognosis and factors affecting the latter for COVID-19-associated SPE. We aimed to describe COVID-19-associated SPE and quantify and compare mortality and factors affecting mortality among the cases. We included a total of 25 publications with a total of 35 cases. The average age was 45 ± 16.3 years with 11 females and 24 males. Dyspnoea (82.5%), orthopnoea (43.5%), and cough (43.5%) were the most common symptoms, and obstructive shock was present in five (21.7%) patients. The average reported oxygen (O_2_) saturation was 85.8% ± 11.9 mm Hg. Hypertension (26.1%), diabetes (21.7%), and deep vein thrombosis (21.7%) were the most commonly reported comorbidities. Right heart strain was recognized in seven (30%) patients on electroencephalogram (S1QIIITIII) and 12 (52.2%) patients on echocardiogram. Anticoagulation, thrombolysis, and percutaneous intervention were tried in 21 (91.3%), 13 (56.5%), and 6 (26.1%) cases, respectively. Despite the aggressive management, 2 of 25 (8.7%) patients died in our smaller case report cohort. We conclude that despite aggressive management modalities, the mortality of SPE remains high in COVID-19.

## 1 Introduction

Saddle pulmonary embolism (SPE) is a large pulmonary embolism (PE) that straddles the bifurcation of the pulmonary trunk and extends into both the left and right pulmonary arteries. It is a rare type of PE that can cause sudden death. Higher incidences of cardiac arrest, cardiogenic shock, respiratory failure, and mean length of stay (LOS) have all been linked to SPE [1]. Consequently, SPE has historically been considered a condition associated with high mortality. PE-related death has been observed in the range of 1–7.0% in non-SPE patients [2]. SPE has been reported to occur in about 2.6–5.4% of all acute PE patients and is expected to predict poorer outcomes if not treated aggressively [3]. Standard anticoagulation (AC), systemic thrombolysis, catheter-directed thrombolysis and surgical embolectomy are all alternatives for the treatment.

Thrombotic events are one of the common features of coronavirus diseases 2019 (COVID-19) [4]. The underlying pathophysiological mechanisms are complex and involve two distinct mechanisms, namely, thromboembolism and immunothrombosis. The former is characterized by the activation of coagulation cascade due to endothelial cell damage, and the latter is characterized by intense inflammatory and immune reactions causing massive coagulation cascade activations and intense and prolonged fibrin degradation [5,6]. However, understanding this association and treating it promptly are critical for effectively managing this condition. The incidence of PE in COVID is known to be around at least 15% [4]. However, the incidence of SPE in COVID-19 is not widely known and needs further studies for its estimation. Some clinical features denoting severity in SPE, such as obstructive shock and right ventricular (RV) strain pattern, are associated with increased mortality [7]. Mortality in COVID-19 itself has been found to be associated with comorbidity status as well as PE [8]. The overall mortality of SPE in COVID-19 patients has also not been studied systematically in larger studies. More studies are needed on SPE in COVID-19 patients to estimate mortality and factors associated.

Cases of SPE in COVID-19 patients have also been reported with conflicting results concerning treatment success. In our recent systematic review of SPE, we reported that SPE mortality was 4.6% and found that AC, surgical thrombectomy, thrombolysis, and percutaneous treatment significantly increased the odds of survival in SPE patients [9]. In this focused systematic review, we aim to describe SPE in the context of COVID-19 and quantify and compare mortality, and possible factors affecting mortality among the cases.

## 2 Materials and methods

### 2.1 Literature search

PubMed, Scopus, and Google Scholar were searched for articles (any date up to February 28, 2022) reporting patients with SPE. Keyword as generated used advanced search function and was (“covid 19”[All Fields] OR “covid 19”[MeSH Terms] OR “covid 19 vaccines”[All Fields] OR “covid 19 vaccines”[MeSH Terms] OR “covid 19 serotherapy”[All Fields] OR “covid 19 serotherapy”[Supplementary Concept] OR “covid 19 nucleic acid testing”[All Fields] OR “covid 19 nucleic acid testing”[MeSH Terms] OR “covid 19 serological testing”[All Fields] OR “covid 19 serological testing”[MeSH Terms] OR “covid 19 testing”[All Fields] OR “covid 19 testing”[MeSH Terms] OR “sars cov 2”[All Fields] OR “sars cov 2”[MeSH Terms] OR “severe acute respiratory syndrome coronavirus 2”[All Fields] OR “ncov”[All Fields] OR “2019 ncov”[All Fields] OR ((“coronavirus”[MeSH Terms] OR “coronavirus”[All Fields] OR “cov”[All Fields]) AND 2019/11/01:3000/12/31[Date - Publication])) AND ((“saddle”[All Fields] OR “saddles”[All Fields]) AND (“pulmonary embolism”[MeSH Terms] OR (“pulmonary”[All Fields] AND “embolism”[All Fields]) OR “pulmonary embolism”[All Fields])). Scopus keywords and google scholar keywords were “COVID-19 AND Saddle Pulmonary Embolism” and “COVID-19 AND Saddle Pulmonary Embolism,” respectively.

### 2.2 Study selection and data extraction

Retrieved articles from the search strategy were uploaded to Rayyan.AI software for screening. HC and FA screened the articles independently. WI conducted an independent review of the disputed articles for a final decision. The extracted studies were initially screened from the title, abstract, and keywords, followed by a full-length screening.

### 2.3 Inclusion criteria

Studies published in English, reporting primary patient data (case reports, series, observational retrospective, prospective studies, and clinical trials) regarding SPE in the context of COVID-19 patients, were added to the systematic review.

### 2.4 Exclusion criteria

Exclusion criteria included studies in languages other than English. In addition, review articles with secondary patient data were excluded.

### 2.5 Quality assessment

FA and HC assessed the quality of the added studies independently. Case reports and series were assessed using the Joanna Briggs Institute case report appraisal checklist for inclusion in systematic reviews [10].

### 2.6 Data collection and analysis

Data on demographics, clinical characteristics, clinical observations, diagnostics results, LOS, management, and outcomes were extracted and analyzed. Data were collected in Microsoft Excel 2016, and analysis was performed in R Studio 2022.2.3. Dplyr, psych, and epitools packages were used for analysis in R Studio. We excluded the case series in the inferential statistics as the individual case data for each case were unavailable in these publications. The chi-square test and Mann-Whitney *U* tests were used where appropriate.


**Systematic Review Registration:** The protocol has been registered in the International Prospective Register of Systematic Reviews (PROSPERO): CRD42021286270. https://www.crd.york.ac.uk/PROSPERO/display_record.php?RecordID=286270.

## 3 Results

**Figure 1:**
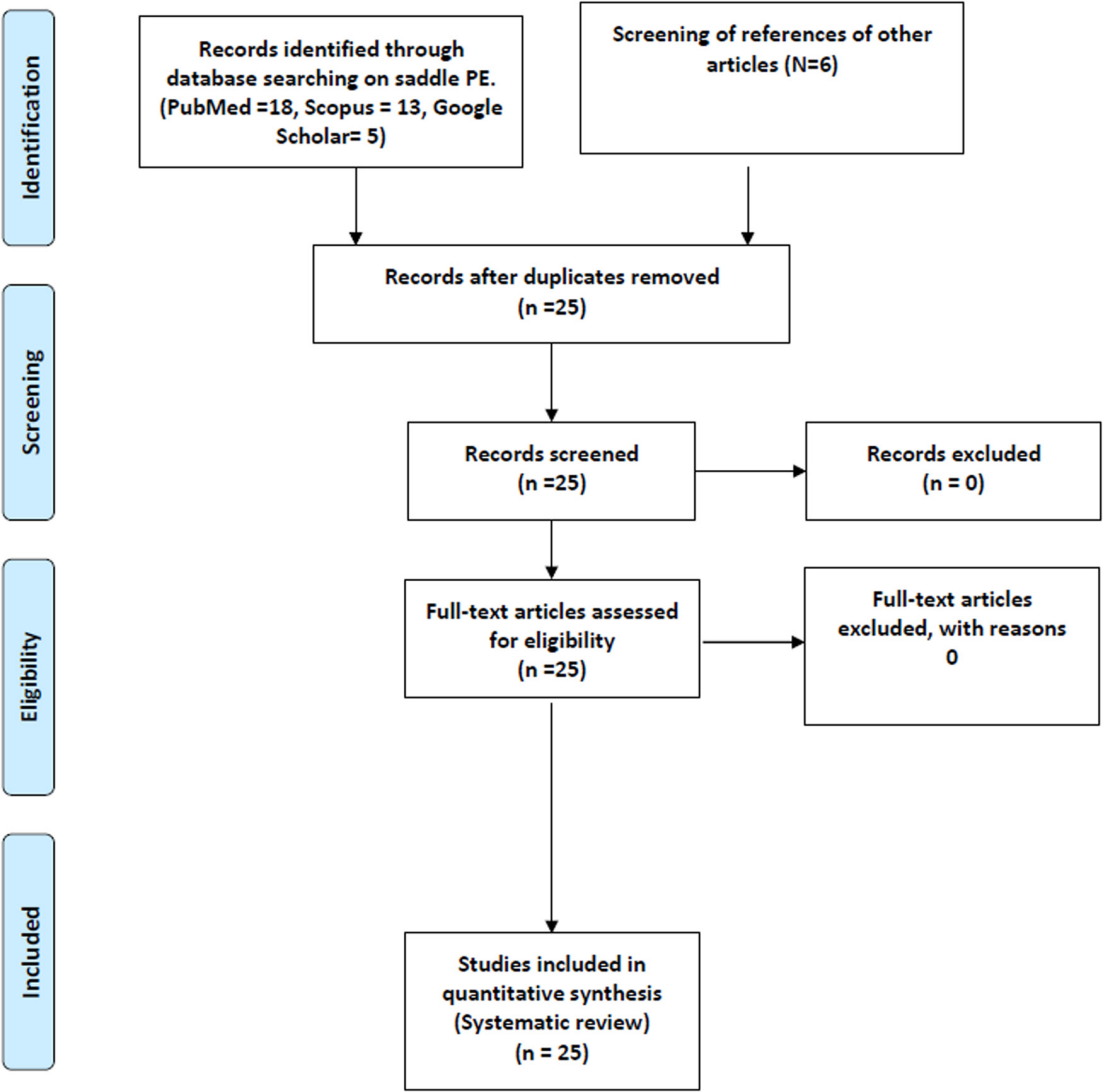
The Prisma flow diagram summarizing the inclusion and exclusion of relevant studies.

A total of 25 publications (case reports and series) were identified with 35 cases (Figure 1). The average age was 45.6 ± 16.3 years, with 11 females (31.4%) and 24 males (68.6%). As discussed in Section 2, we included only the case reports for our descriptive and inferential analysis of clinical parameters, observations, diagnostic testing, treatment, and outcome. COVID-19 variant information was not available for any of the publications. All of our included cases, however, correspond to a time period from summer of 2020 to mid-summer of 2021. Hence, it is probable that alpha and delta strains were the most prevalent ones.

Only four publications mentioned prophylactic AC, despite which patients developed the PE [11–13]. Himwaze et al. in the autopsy study mentioned four patients being on AC prophylactically and still having the thrombotic event [11]. None, except one of the cases, Shazley et al. mentioned COVID-19 vaccination [14].

The basic characteristics of the patients, including clinical presentations, investigations, and treatment modalities, are all listed in Table 1. Dyspnoea (82.5%), orthopnoea (43.5%), and cough (43.5%) were the most common symptoms. Features of obstructive shock were reported in 5 (21.7%) patients. The average reported oxygen (O_2_) saturation was 85.8% ± 11.9. It was normal (≥94%) only in 2 of 14 patients (14.3%) and <90% in 6 of 14 (42.8%) cases.

**Table 1:** Clinical characteristics and outcomes of patients with SPE in the setting of COVID-19 infection (*N* = 35)

	Gender	Females: 11 (31.4%)
		Male: 24 (68.5%)
	Age	Mean: 45 years
		SD: 16.3
Clinical presentations	Syncope	4/23 (14.7%)
	Dyspnoea	19/23 (73%)
	Chest pain	7/23 (29.4%)
	Hemoptysis	2/23 (5.9%)
	Cough	10/23 (29.4%)
	Orthopnoea	10/23 (41.2%)
	Unconscious on presentation	0
	Lower limb pain	1 (5.9%)
	Obstructive shock	5 (14.7%)
OBS	Systolic blood pressure	Mean: 110.9 ± 26.3 mm Hg
	HR	Mean: 129 ± 18.6
	O2 saturation	85.8 ± 11.8%
Comorbidities	Patent foramen ovale	3/23 (13.0%)
	Any valvular disorder	2/23 (8.7%)
	Diabetes	5/23 (21.7%)
	Hypertension	6/23 (26.1%)
	Lung disease	3/23 (13.0%)
	Chronic kidney disease	1/23 (4.3%)
	Malignancy	1/23 (4.3%)
	Any comorbidity	10/23 (43.5%)
	DVT	5/23 (21.7%)
	History of PE	1/23(4.3%)
Diagnostics	ECG findings	Normal/not mentioned: 9 (39.1%)
		Right heart strain (S1Q3T3): 7 (30.4%)
		Other findings: 7 (30.4%)
	Echocardiogram findings	Right ventricular strain: 12 (52.2%)
		Normal/not mentioned: 11 (47.8%)
	Right heart strain (ECG or echo)	Present: 16 (69.6%)
		Not present or mentioned: 7 (30.4%)
	CTPA	Saddle thrombus: 20 (86.9%)
		Other findings: 3 (13.1%)
Treatments employed	Surgical removal	0
	Catheter removal	6 (26.1%)
	Thrombolysis	13 (56.5%)
	Anticoagulation	21 (91.3%)
	IVC filter	1 (4.3%)
Outcome	LOS	Mean: 9.8 days
		SD: 8.9
	ICU admissions	15/23 (65.2%)
	Outcome (total)*	Alive: 21 (61.8%)
		Died: 13 (38.2%)
		Total: 33 (100%)
	Outcome (case reports)	Alive: 21 (91.3%); died: 2 (8.7%)
		Total: 23 (100%)

^*^Total outcome includes autopsy studies where all the patients were deceased.

IVC: inferior vena cava; SD: standard deviation; HR: heart rate; LOS: length of stay; ICU: intensive care unit; CTPA: computed tomography pulmonary angiogram; ECG: electrocardiogram; Echo: echocardiogram; DVT: deep vein thrombosis; PE: pulmonary embolism; O2: oxygen.

The most common comorbidities were hypertension present in 6 (26.1%) and diabetes mellitus in 5 (21.7%) cases, followed by lung disease and patent foramen ovale (PFO) in 3 (13.0%) cases each. Deep vein thrombosis (DVT) was reported in five cases (21.7%), with two being bilateral and three unilateral.

Right heart strain pattern on electroencephalogram (ECG) (S1QIII TIII) was identified in seven (30%) patients, while another seven (30%) cases had abnormal ECG with findings other than the RV strain pattern. Echocardiography revealed RV strain in 12 (52.2%) patients. Initial computed tomography pulmonary angiogram (CTPA) revealed SPE in 20 (86.9%) of cases, whereas 8 cases mentioned additional non-SPEs upon imaging.

The average hospital stay was 9.8 ± 8.9 days, with 15 reported admissions to the intensive care unit (ICU). The most common treatment modality was AC, reported in 21 (91.3%) cases, while thrombolysis and percutaneous thrombectomy percutaneous intervention (PCI) were tried in 13 (56.5%) and 6 (26.1%) cases, respectively. Alteplase (r-tpa) and tPA were two reported thrombolytic agents used in six and four cases, respectively. An inferior vena cava filter was reported in one case for recurrent thromboembolism.

The outcome was 2 of 21 (8.7%) deaths in our smaller case report cohort.

### 3.1 Inferential statistics

Statistical analysis was performed to assess the relationship of outcome with gender, age, clinical presentations, comorbidity status, and diagnostic findings and treatment. We found no statistical difference between any of the variables mentioned earlier in terms of outcome (*p* > 0.05), as presented in Table 2. Comorbidity status (defined as the presence of either of the following: hypertension, diabetes, chronic kidney disease, lung disease, any valvular disorder, PFO, or history of PE) was also unrelated to the outcome (*P* = 0.34). The mean age of alive and dead was 45.7 and 61.0 years, respectively, but the difference was statistically insignificant (*P* = 0.23). Gender also did not influence the outcome in our small cohort (*P* = 1).

**Table 2:** Inferential statistics of patients with SPE and COVID-19 based on mortality (*N* = 24)

Variable	Alive (21) vs dead (2)	*P* value
Gender: male vs female	15 vs 1 6 vs 1	1
Syncope	4 vs 0	1
Dyspnea	17 vs 2	1
Chest pain	7 vs 0	0.86
Hemoptysis	2 vs 0	1
Cough	8 vs 2	0.35
Orthopnea	10 vs 0	0.58
Lower limb pain	1 vs 0	1
Obstructive shock	5 vs 0	—
SBP (mm of Hg)	106 vs 174	0.20
O_2_ saturation	88 vs 57	0.12
Any valvular disorder	2 vs 0	1
Diabetes	4 vs 1	0.90
Hypertension	4 vs 2	0.09
Lung disease	2 vs 1	0.60
Any comorbidity	8 vs 2	0.35
ECG (RV strain)	7 vs 0	0.86
Echo (RV strain)	10 vs 2	0.49
CTPA (SPE)	19 vs 2	1
PCI	4 vs 2	0.09
Thrombolysis	13 vs 0	0.3
Anticoagulation	19 vs 2	1
IVC filter	1 vs 0	1

PCI: percutaneous intervention, IVC filter: inferior vena cava filter, SBP: systolic BP; RV strain: right ventricular strain; SPE: saddle pulmonary embolism.

## 4 Discussion

Historically, SPE has been reportedly associated with a higher (9.2–65%) in-hospital mortality in multiple studies [1,15] compared with a relatively lower overall mortality of non-SPE cases, which varies from 0.2 to 6% depending on the clinical context [16–20]. Pathak et al. compared outcomes of hospitalizations due to PE in the United States and reported that SPE represented only 0.16% of all PE-related hospitalizations [21]. They reported similar outcomes but higher rates of cardiogenic shock, respiratory failure, thrombolysis, and LOS in the SPE group. Multiple comparative studies have reported similar outcomes in SPE and non-SPE [3,22].

To the best of our knowledge, our study represents the first systematic review of SPE in COVID-19 cases, charting the prevalence of mortality and possible prognostic factors. Our recent systematic review discussed the prognostic factors among SPE cases and found an overall mortality of 4.6% [9]. We report a slightly higher mortality of 8.7% in our current small cohort of patients with SPE in the context of COVID-19, excluding the post-mortem studies for apparent bias.

PE is risk stratified for management decisions and prognostic determination using the PE severity index (PESI), a widely validated risk score [23]. This scale has historically been based on epidemiological variables (age and gender), comorbidity status (cancer, heart failure, and lung failure), as well as vital instability [7]. A simplified PESI later on excluded gender and some of the vitals [24]. In terms of clinical severity, it is classified into high risk (massive), intermediate risk (submassive), and low risk based on hemodynamic instability, demonstration of RV dysfunction on echocardiogram or CTPA, PESI score, and elevated troponins. There is a significant difference in outcomes between these severity classes with massive PE mortality ranging from 20 to 65% and submassive with 5–25% [1,25]. Low-risk PE has been reported to carry a mortality risk of close to 1%. Although hemodynamic instability was not a common feature for most of our patients, RV dysfunction on Echo and Saddle embolus on CTPA was demonstrable in 70 and 86% of patients in our cohort, respectively (Table 1). Almost all of our cases constitute an intermediate risk, as all had a CTPA elucidated defect straddling the bifurcation of vessels, with the exception of five (14%) who presented with hemodynamic instability.

Two of our case series were post-mortem and represented 12 patients in our cohort [11,26]. Mucheleng’anga et al. described 21 cases in a post-mortem case series of COVID-19 patients from Zambia, Africa [26]. They mentioned eight (38%) cases with SPE among their cohort, while details were given for 7, which we eventually included in this study. PE in one form or another (shower, saddle, non-saddle) was diagnosed on autopsy in 17 patients (81%), while the remaining were found to have changes consistent with severe pneumonia. The authors later from the same region presented another case series of 29 cases with 5 (17%) cases of SPE. Because of the nature of these studies and the type of data presented, it was not possible to include these cases in the inferential statistics (Table 3).

**Table 3:** Availabl**e** clinical details of all 25 included studies

Sr no.	Author	Epidemiology, presentations	Comorbidities	Investigations	Treatment	Outcome
**1**	Cristoforo et al. [31]	11 M	Nephrotic syndrome	Diagnosed on CT, ECG: RV strain		Discharged
**2**	Teklie et al. [32]	20 M		Elevation of 50% of complement	Heparin infusion, bilateral PA catheter	Discharged
**3**	Molina et al. [33]	23 M	Nitric oxide inhalation	*ECG*: Sinus tachy, RBBB, ECHO: RV strain, B/L DVT	Thrombolysis (tPA)	Alive
**4**	Atallah et al. [30]	29 M	Autism	ICU MV, CT angio	Thrombolysis (tPA)	Discharged
**5**	Kharazmi et al. [34]	32 M	PFO	Echo: RVS, TIT, IVC clot	Thrombolysis (r-tPA)	Discharged
**6**	Hoilat et al. [35]	32 M		CT angiography	ICU MV, mechanical embolectomy	Discharged
**7**	Vyas et al. [36]	32 M		CT angiography	Mechanical thrombectomy	
**8**	Himwaze et al. [11]	33 M with dyspnoea and fever	HIV			Died (Autopsy)
**9**		36 M dyspnoea/Abdo pain, abdominal TB				Died (Autopsy)
**10**		47 F saddle emboli, heavy lungs (>1,000 g each),		Deep venous thrombosis, and diffuse alveolar damage		Died (autopsy)
**11**		65 M	CVA			Died (autopsy)
**12**		70 F	Hypertension	DVT, thrombosed mesenteric arteries		Died (autopsy)
**13**	Pendower et al. [37]	64 F syncope, SOB, dizziness	Old DVT	Echo: RVS.	Infusion catheter (alteplase)	Discharged
				CT Angio,		
**14**	Flemming et al. [38]	47 M		*ECG*: Normal	Catheter thrombolysis	Discharged
				*Echo*: RVS, CT angio		
**15**	Jafari et al. [39]	50 F saddle after 3 days stay in hospital		CTPA, AC		Discharged
**16**	Ali et al. [40]	52 F SPE 7 days after discharge		*ECG*: S1Q3T3	r-tPA	Discharged
				CTPA syncope	ICU	
					Anticoagulation (enoxaparin)	
**17**	Shazley and Alshazley [14]	52 M after J&J vaccine	Hypertension, diabetes, obesity	Bilateral DVT CTPA	ICU MV	Death in 1 month
					PCI	
**18**	Valencia-Manrique et al. [12]	52 F	Diabetes, obesity	S1Q3T3	ICU, anticoagulation (enoxaparin)	
**19**	Ismail et al. [41]	52 M with left weakness	DVT	ECG: bifascular block,	Anticoagulation (IV heparin)	Recovered
				Echo: RV embolus, third-degree AV block,		
20	Yu et al. [42]	55 M syncope	Hypertension, diabetes	Echo: intracardiac shunt, CTPA	Catheter-directed thrombolysis	Discharged
					(t-PA), Anticoagulation (IV heparin)	
**21**	Aaron et al. [43]	56 M	DVT	Echo: RV strain CTPA	ICU MV	Discharged
					tPA (resolution) Saddle PE again in 1 week: tPA	
**22**	Khurram et al. [44]	61 M	End-stage renal disease, OSA, diabetes, CVA, BPH, superior ophthalmic vein thrombosis	CTPA ICU	Anticoagulation (LMWH)	Discharged
**23**	Bhatt et al. [45]	65 M DVT	DVT	CTPA	Anticoagulation (IV heparin)	Discharged
**24**	Aoi et al. [13]	70 F	Hypertension, DM, history of SVT	ECG: S1Q3T3	PCI + Cath thrombolysis	Died
				Echo: RVS		
				CTPA: CIT		
**25**	Fujikura et al. [46]	77 F DVT, discharged → missed apixaban → dyspnea again	CVA, DM, PFO, DVT, history of cancer	Echo: RV strain.	Anticoagulation (IV heparin)	Discharged
				CTPA		
				Re-echo: B/l atrial RA CIT		
**26**	Chang and Segura [47]	43 M		CTPA	ICU MV	Discharged
					tPA	
					Cardiac support	
**27**	Nehme et al. [48]	56 M		CTPA multiple Pes, tracheal necrotic flap	Thrombolysis (alteplase)	Discharged
					ICU MV	
					VAP	
**28**	Namburu et al. [49]	69, ST elevation, Takasubo cardiomyopathy		*Cath*: Takasubo Cardiomyopathy	Thrombolysis	Discharge
				*Echo*: Enlarged right ventricles, atrial thrombus, CTPA		
**29**	Mucheleng’anga et al. [26]	32 M Headache and diarrhea				Died
30		37 M chest pain dyspnoea				Died
**31**		51 M Chest pain and dyspnoea				Died
**32**		20 M Dyspnea and painful legs				Died
**33**		39 F Chest pain				Died
**34**		38 M				Died
**35**		25 F Convulsions DVT				Died

DM: diabetes mellitus CVA: cerebrovascular accident, DVT: deep venous thrombosis, CTPA: CT pulmonary angiogram, MV: mechanical ventilation, VAP: ventilation associated pneumonia, Cath: catheterization (Coronary), RV: right ventricle, BPH: benign prostatic hyperplasia, SVT: supraventricular tachycardia, TB: tuberculosis, SOB: shortness of breath.

The proportion of SPE among PE patients has largely been found to be 2–5% [15]. The two autopsy studies we included describe the share of SPE to be ranged from 17 to 38% among all patients who died of COVID [11,26]. It would be prudent to keep in mind the nature of these two studies, which included only the COVID patients who died and hence likely to overestimate the SPE proportion. Similarly, prophylactic AC, which was rare during the first wave of the COVID-19 pandemic and became commonplace later, is also very likely to affect this number [27].

In one of the interesting studies on COVID and PE, Hobohm et al. reported a 1.9% prevalence of PE in hospitalized patients with COVID-19. More than 33% of COVID patients with PE had ICU admission. They also reported a significantly higher case fatality rate in COVID patients with PE vs COVID without PE (28.7% vs 17.7%). Another interesting trend was higher case fatality in COVID and PE patients versus patients with PE without COVID (28.7% vs 12.5%) [8]. In the current systematic review, ICU admission was reported for 15 (76% of smaller cohort) cases, which is much higher but in line with the clinical instability and interventions required for this subset of PE (saddle).

The pathophysiology of PE in COVID is complex and multifactorial. The distinct angiocentric feature of COVID-19 was recognized relatively early in the pandemic [28]. Two distinct mechanisms have been elucidated for the overall pathogenesis of thrombosis in COVID-19. The first mechanism is the classic thromboembolism that arises due to sepsis and endothelial dysfunction, leading to tissue factor expression, thrombosis cascade activation, and finally classic thromboembolism [6]. The second mechanism, somewhat novel, involved micro-thrombosis due to the activation of immune pathways causing severe organ damage in lungs, kidneys, skin (blue toes), and other organs [29]. The latter severe type causing widespread immune-mediated microthrombi was reported to be found in patients on prophylactic AC. We found that three of the publications (with a total of six patients) mentioned PE despite prophylactic AC [11,13,30]. PE, one of the sequelae of the angiocentric activity of the disease, has been found in most COVID-19 patients upon autopsy. The prevalence of any pulmonary thromboembolism in the two autopsy studies in our cohort was 80.9 and 48.3% among those deceased [11,26]. It was also one of the leading causes of death among those.

Treatment modalities in our sample did not affect the outcomes. We believe that this is because of the small number in one group, i.e., the deceased. Our general SPE study earlier recognized that all interventions affected mortality in the SPE. However, we are unsure if this is due to forward or reverse causation, i.e., the severity of SPE (upon presentation) discouraging operators or not allowing enough time for clinicians to perform any intervention [9]. Undoubtedly more studies, clinical trials, in particular, are needed to look at the impact of different treatment modalities on the recovery and outcome of SPE in COVID patients.

Our study has several limitations. The small sample size of our study makes our data less reliable, as the statistical inferences are not generalizable. The types of studies included (case reports and series) and the cases they represented suffer multiple biases, including publication and survival ones. These studies may be a source of selection bias in our sample, something inherent for most systematic reviews, which include these types of studies. Barring the aforementioned limitations, we believe our study is free from any systematic biases. Nevertheless, this is the first systematic review highlighting an important and clinically significant combination of SPE with COVID-19 infection and opening doors to further research on the topic.

## 5 Conclusion

SPE is relatively common in COVID-19 cases and is associated with high mortality. More extensive data are needed to understand the association between COVID-19 infection and SPE.

## Supplementary Material

Supplementary material
