# Supplementary material for: Saddle pulmonary embolism in the setting of COVID-19 infection: A systematic review of case reports and case series
Source: Open Med (Wars). 2023 Jun 1;18(1):20230724. doi: 10.1515/med-2023-0724 (PMC10238810; doi:10.1515/med-2023-0724)
Supplement: Supplementary material [file med-2023-0724-sm.pdf]

Studies added in the systematic review (1-25)

1. Aaron L, Welch M, Shah A, Thomas T, McKechnie SR. Recurrent massive pulmonary emboli in a critically ill patient with COVID-19. Anaesthesia reports. 2020;8(2):94-7.
2. Ali S, Mathew S, Pappachan JM. Acute cor pulmonale from saddle pulmonary embolism in a patient with previous COVID-19: should we prolong prophylactic anticoagulation? International journal of infectious diseases : IJID : official publication of the International Society for Infectious Diseases. 2020;97:299-302.
3. Aoi S, Kakkar AM, Golowa Y, Grushko M, Coyle CM, Elrafei T, et al. Saddle pulmonary embolism and clot in transit in COVID-19 infection: a case report of catastrophic venous thromboembolism. European Heart Journal - Case Reports. 2020;4(6):1-6.
4. Ataallah B, Sharma A, Tamanna S, Ng J, Haggerty G. Major Thrombotic Event Despite Anticoagulation in a Patient With COVID-19. Cureus. 2020;12(6).
5. Bhatt H, Singh S. Venous thromboembolism and COVID-19: a case report and review of the literature. Journal of Medical Case Reports. 2020;14(1):1-4.
6. Chang EE, Segura EMBA. Acute Refractory Hypoxemia Due to Pulmonary Saddle Embolism in a COVID-19 Patient with ARDS. Past Present and Future and Review of Literature. 2021.
7. Cristoforo T, McKinley G, Ambrosio P. Saddle pulmonary embolism in a pediatric patient with nephrotic syndrome and recent COVID-19 pneumonia: A case report. The American journal of emergency medicine. 2021;48:376.e1-.e2.
8. Flemming N, Sittol R, Simmonds R-K, Grant J, Lofters J, Alfaki M, et al. SADDLE UP! A CASE OF COVID-19-ASSOCIATED SADDLE PULMONARY EMBOLISM. Chest. 2020;158(4):A1017-A.
9. Fujikura K, Fontes JD, Taub CC. Saddle pulmonary embolism and thrombus-in-transit straddling the patent foramen ovale 28 days after COVID symptom onset. Echocardiography (Mount Kisco, NY). 2020;37(8):1296-9.
10. Himwaze CM, Telendiy V, Maate F, Mupeta S, Chitalu C, Chanda D, et al. Post-mortem examination of Hospital Inpatient COVID-19 Deaths in Lusaka, Zambia - A Descriptive Whole-body Autopsy Series. International journal of infectious diseases : IJID : official publication of the International Society for Infectious Diseases. 2021;108:363-9.
11. Hoilat GJ, Durer C, Durer S, Gupta P. Percutaneous Mechanical Pulmonary Thrombectomy in a Patient With Pulmonary Embolism as a First Presentation of COVID-19. Cureus. 2020;12(8).
12. Ismail Z, Salabei JK, Stanger G, Asnake ZT, Frimer L, Smock A. Third-Degree Heart Block Associated With Saddle Pulmonary Embolism: A Rare Sequelae of COVID-19-Induced Hypercoagulable State. Cureus. 2021;13(7).
13. Jafari R, Cegolon L, Jafari A, Kashaki M, Otoukesh B, Ghahderijani BH, et al. Large saddle pulmonary embolism in a woman infected by COVID-19 pneumonia. European heart journal. 2020;41(22):2133-.
14. Kharazmi A, Mirbaha S, Hatamabadi H, Shojaeian F, Omidi F. Medical treatment for paradoxical and saddle pulmonary embolism in a young man with patent foramen ovale and coronavirus disease 2019. Turkish Journal of Emergency Medicine. 2021;21(3):133-.
15. Khurram R, Naidu V, Butt MF, Durnford L, Joffe M. Superior ophthalmic vein thrombosis secondary to COVID-19: an index case. Radiology case reports. 2021;16(5):1138-43.
16. Molina MF, Al Saud AA, Al Mulhim AA, Liteplo AS, Shokoohi H. Nitrous oxide inhalant abuse and massive pulmonary embolism in COVID-19. The American journal of emergency medicine. 2020;38(7):1549.e1-.e2.

17. Mucheleng'anga LA, Telendiy V, Hamukale A, Shibemba AL, Zumla A, Himwaze CM. COVID-19 and Sudden Unexpected Community Deaths in Lusaka, Zambia, Africa - A Medico-Legal Whole-Body Autopsy Case Series. International Journal of Infectious Diseases. 2021;109:160-7.
18. Namburu L, Bhogal SS, Ramu VK. COVID-19-Induced Takotsubo Cardiomyopathy With Concomitant Pulmonary Embolism. Cureus. 2021;13(10):e18693.
19. Nehme R, Fleifel M, Abou Khalil M, Al Dailaty A. A case of massive saddle pulmonary embolism and benign tracheal stenosis in a patient with COVID-19 infection. Respirology case reports. 2021;9(11).
20. Pendower L, Benedetti G, Breen K, Karunanithy N. Catheter-directed thrombolysis to treat acute pulmonary thrombosis in a patient with COVID-19 pneumonia. BMJ case reports. 2020;13(8).
21. Shazley O, Alshazley M. A COVID-Positive 52-Year-Old Man Presented With Venous Thromboembolism and Disseminated Intravascular Coagulation Following Johnson & Johnson Vaccination: A Case-Study. Cureus. 2021;13(7).
22. Valencia-Manrique JC, Ghosh K, Velasquez Espiritu MR, Poor A. A Case of Saddle Pulmonary Embolism in the Recovery Phase of COVID-19 Infection. American Thoracic Society International Conference Meetings Abstracts American Thoracic Society International Conference Meetings Abstracts. 2021:A3501-A.
23. Vyas V, Kanagalingam G, Yadava S, Gambhir HS, Costanza M, Chaudhuri D. Bilateral pulmonary artery thrombectomy with saddle embolism and COVID-19 infection. Proceedings (Baylor University Medical Center). 2020;33(4):666-7.
24. Y T, V C, J G, R V. Role Of Classical Complement Activation In SARS-Cov-2 Infection; A Case Report Of COVID-19 In A Patient With Saddle Pulmonary Embolism. J Med Case Rep Case Series. 2020;1(4).
25. Yu MD, Desai N, Sanagala T, Darki A. Paradoxical Embolism Causing Myocardial Infarction in a COVID-19 Patient Presenting With Pulmonary Embolism. Cureus. 2021;13(3).
